# Correction: Pharmacophore-based approaches in the rational repurposing technique for FDA approved drugs targeting SARS-CoV-2 M^pro^

**DOI:** 10.1039/d0ra90120b

**Published:** 2020-11-25

**Authors:** Vishal M. Balaramnavar, Khurshid Ahmad, Mohd Saeed, Irfan Ahmad, Mehnaz Kamal, Talha Jawaid

**Affiliations:** Department of Medicinal and Pharmaceutical Chemistry, Global Institute of Pharmaceutical Education and Research Jaspur Road Kashipur 244713 India v.balaramnavar@gmail.com; Department of Medical Biotechnology, Yeungnam University Gyeongsan 38541 South Korea; Department of Biology College of Sciences, University of Hail Saudi Arabia; Department of Clinical Laboratory Science, College of Applied Medical Sciences, King Khalid University Abha Saudi Arabia; Research Center for Advanced Materials Science, King Khalid University Abha Saudi Arabia; Department of Pharmaceutical Chemistry, College of Pharmacy, Prince Sattam Bin Abdulaziz University P.O. Box No. 173 Al-Kharj 11942 Kingdom of Saudi Arabia; Department of Pharmacology, College of Medicine, Al Imam Mohammad Ibn Saud Islamic University (IMSIU) Riyadh 13317 Saudi Arabia

## Abstract

Correction for ‘Pharmacophore-based approaches in the rational repurposing technique for FDA approved drugs targeting SARS-CoV-2 M^pro^’ by Vishal M. Balaramnavar *et al.*, *RSC Adv.*, 2020, 10, 40264–40275, DOI: 10.1039/D0RA06038K.

The authors regret that the name of one of the authors (Talha Jawaid) was shown incorrectly in the original article. The corrected author list is as shown above.

The Royal Society of Chemistry apologises for these errors and any consequent inconvenience to authors and readers.

## Supplementary Material

